# Downregulation of HLA Class I Renders Inflammatory Neutrophils More Susceptible to NK Cell-Induced Apoptosis

**DOI:** 10.3389/fimmu.2019.02444

**Published:** 2019-10-15

**Authors:** Elin Bernson, Karin Christenson, Silvia Pesce, Malin Pasanen, Emanuela Marcenaro, Simona Sivori, Fredrik B. Thorén

**Affiliations:** ^1^TIMM Laboratory, Sahlgrenska Cancer Center, University of Gothenburg, Gothenburg, Sweden; ^2^Department of Infectious Diseases, Institute of Biomedicine, University of Gothenburg, Gothenburg, Sweden; ^3^Department of Oral Microbiology and Immunology, University of Gothenburg, Gothenburg, Sweden; ^4^Department of Experimental Medicine, University of Genoa, Genoa, Italy; ^5^Center of Excellence for Biomedical Research (CEBR), University of Genoa, Genoa, Italy

**Keywords:** neutrophil, NK cell, HLA class I, immunoregulation, neutrophil apoptosis

## Abstract

Neutrophils are potent effector cells and contain a battery of harmful substances and degrading enzymes. A silent neutrophil death, i.e., apoptosis, is therefore of importance to avoid damage to the surrounding tissue and to enable termination of the acute inflammatory process. There is a pile of evidence supporting the role for pro-inflammatory cytokines in extending the life-span of neutrophils, but relatively few studies have been devoted to mechanisms actively driving apoptosis induction in neutrophils. We have previously demonstrated that natural killer (NK) cells can promote apoptosis in healthy neutrophils. In this study, we set out to investigate how neutrophil sensitivity to NK cell-mediated cytotoxicity is regulated under inflammatory conditions. Using *in vitro*-activated neutrophils and a human skin chamber model that allowed collection of *in vivo*-transmigrated neutrophils, we performed a comprehensive characterization of neutrophil expression of ligands to NK cell receptors. These studies revealed a dramatic downregulation of HLA class I molecules in inflammatory neutrophils, which was associated with an enhanced susceptibility to NK cell cytotoxicity. Collectively, our data shed light on the complex regulation of interactions between NK cells and neutrophils during an inflammatory response and provide further support for a role of NK cells in the resolution phase of inflammation.

## Introduction

Neutrophils are innate immune cells that play a key role in the defense against invading microbes. In circulation, neutrophils are in a resting state, but in response to proinflammatory signals they become alerted, extravasate and migrate toward the site of damage or infection. Neutrophils contain numerous intracellular granules containing pre-formed proteins needed to execute their functions. Thus, when neutrophils are stimulated, they rapidly mobilize specific types of granules to equip the cell with new surface structures and to release soluble factors to the extracellular space (1). The transmigration process *per se* is dependent on degranulation, which enables attachment to endothelial cells, and subsequently chemotaxis-driven migration (2). However, the rearrangement of surface structures does not only involve granule mobilization, but also cleavage or internalization of surface structures; CD62L, involved in attachment to endothelial cells, is for example shed from the neutrophil surface already in circulation (3).

Neutrophils have a rich arsenal of toxic substances and degrading enzymes stored in their granules, which are used to eradicate an ingested prey. The toxic content makes neutrophils a potential danger to the surrounding tissue and it is thus of importance that neutrophils are removed from the inflammatory site after they have fulfilled their tasks. A tightly controlled death process where the cell is degraded from the inside while the surface membrane remains intact, is therefore of importance for termination of the inflammatory process (4). Neutrophil apoptosis can be induced by interaction with other immune cells inducing pro-apoptotic signaling *via* death receptors on the neutrophil surface (5); e.g., we have previously reported that interaction between NK cells and neutrophils can promote neutrophil apoptosis (6).

Natural killer (NK) cells are cytotoxic cells that can kill aberrant cells without prior sensitization (7). Besides their undisputed role in the defense against viral infections and certain malignancies, a growing body of evidence points to a role for NK cells in immune regulation, both as an early source of cytokines but also by selective killing of immune cells (6, 8–13). The result of an encounter between an NK cell and a target cell is determined by the balance between signals originating from inhibitory and activating receptors expressed on the NK cell surface; thus, the presence of cognate ligands to NK cell receptors (NKRs) on the potential target cell determines the outcome of an NK cell—target cell interaction. When the inhibitory signaling is decreased, or the activating signaling increased, the NK cell cytotoxic machinery may be activated, resulting in release of cytotoxic granules into the immunological synapse. Among the major activating receptors are the natural-killer group 2, member D (NKG2D), recognizing MICA and MICB and different ULBPs; DNAX accessory molecule-1 (DNAM-1), recognizing polio virus receptor (PVR) and Nectin-2; 2B4 recognizing CD48; and the group of natural cytotoxicity receptors (NCRs; NKp30, NKp44, and NKp46), which in part constitute orphan receptors (14, 15), while NKp30 recognizes B7-H6 and BAG-6 (16, 17). The main inhibitory receptors are the inhibitory killer immunoglobulin-like receptors (iKIRs) and NKG2A/CD94 that bind to specific human leukocyte antigen (HLA) class I molecules on target cells. HLA-C binds to the KIR2DL receptors, while certain HLA-B and HLA-A molecules contain a Bw4 motif that is recognized by KIR3DL1. The NKG2A/CD94 heterodimer binds to the non-classical HLA-E molecule, which selectively presents the leader peptides of classical HLA molecules and thus reflects the overall expression of HLA class I in a cell. In addition, the inhibitory receptor LILRB1 recognizes HLA class I molecules. Moreover, HLA class I molecules may also bind to activating NK cell receptors, where the NKG2C/CD94 heterodimer binds to HLA-E, and activating KIRs recognize certain motifs on classical HLA class I molecules. The HLA class I molecules can be up- or downregulated from the cell surface in response to cellular signaling, and free soluble HLA class I molecules has been described in serum, either secreted from cells or shed from the cell surfaces due to proteolytic cleavage (18–20).

As mentioned above, NK cells have been ascribed immunomodulatory functions (21, 22) and previous work from our group has demonstrated that NK cells induce apoptosis in healthy neutrophils in an NCR- and Fas-dependent manner (6). Moreover, in a human *in vivo* blister model of sterile inflammation, NK cell entry into blisters coincided with the appearance of apoptotic neutrophils, suggesting a role for NK cells in terminating an inflammatory response (6). With this background, we hypothesized that modulation of neutrophil surface expression of NKR ligands upon neutrophil activation may lead to altered sensitivity to NK cell-induced apoptosis. In this study, we demonstrate that inflammatory neutrophils, both *in vitro*-stimulated blood neutrophils and *in vivo*-transmigrated neutrophils collected from skin chamber exudates, are more susceptible to NK cell-mediated apoptosis, and that the enhanced sensitivity is associated with a pronounced downregulation of neutrophil HLA class I expression.

## Results

### Surface Expression of HLA Class I Molecules Is Decreased Upon Neutrophil Activation

**Figure 1:**
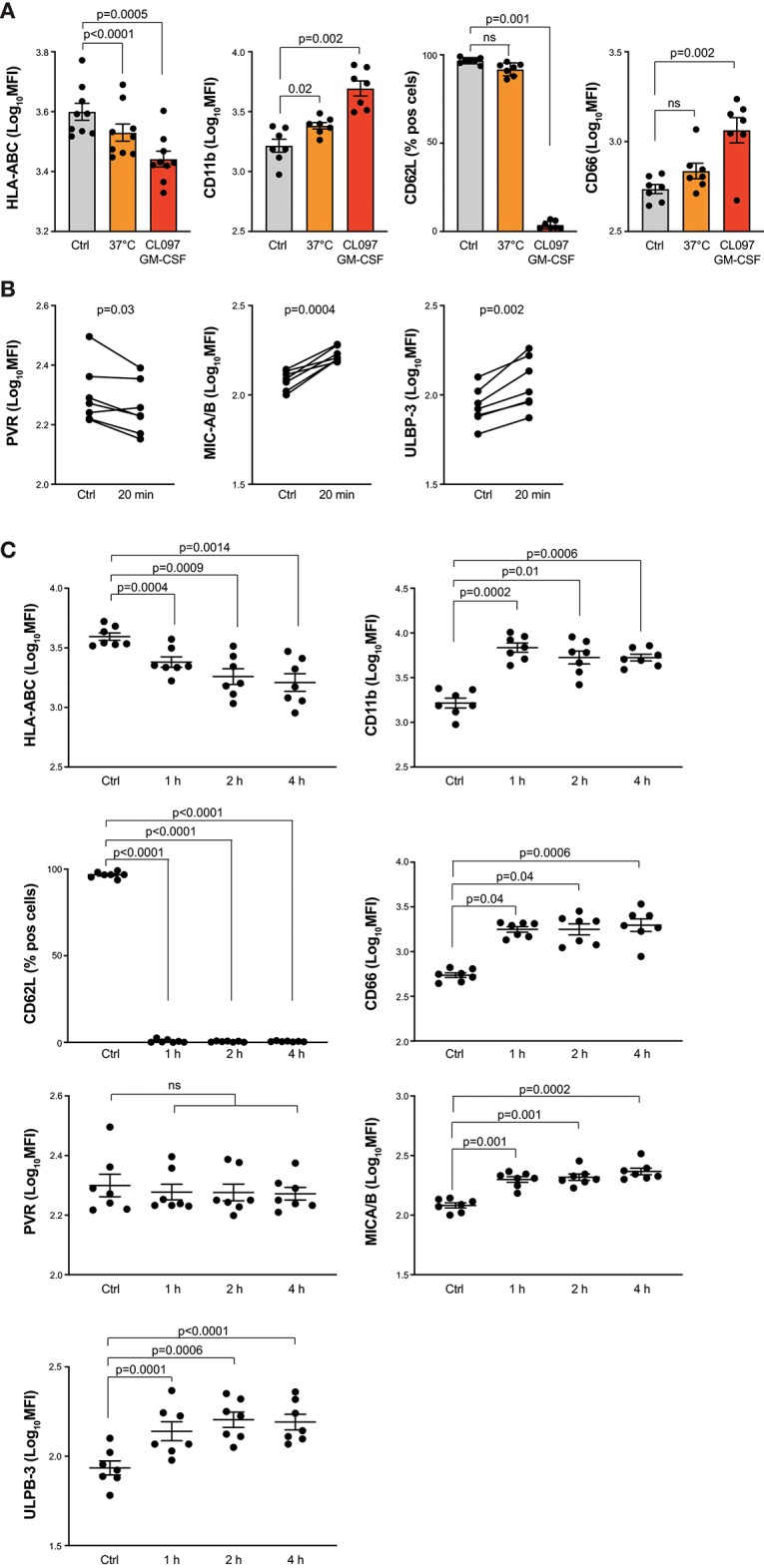
NKR ligand expression on *in vitro*-activated neutrophils. **(A)** Surface expression of HLA-ABC, CD11b, CD66 or CD62L as indicated, on resting neutrophils kept on ice (ctrl) or *in vitro*-activated neutrophils stimulated for 20 min at 37°C alone or in presence of the TLR-agonist CL097 and GM-CSF (one-way ANOVA followed by Dunnett's multiple comparisons test). **(B)** Surface expression of indicated ligands to NKRs on resting neutrophils (ctrl) or *in vitro*-activated neutrophils stimulated for 20 min at 37°C with CL097/GM-CSF (paired *t*-test). **(C)** Expression of NKR ligands and neutrophil degranulation markers on resting neutrophils (ctrl) or *in vitro*-activated neutrophils stimulated for 1, 2, or 4 h at 37°C with CL097/GM-CSF (one-way ANOVA followed by Dunnett's multiple comparisons test). Error bars represent SEM.

NK cell cytotoxicity is triggered when the signals from activating receptor-ligand interactions overcome the inhibitory signals from inhibitory receptors. Thus, in a first set of experiments we investigated to what extent activated neutrophils modulate their expression of ligands to NKRs. In *in vitro* experiments, we combined GM-CSF with the TLR7/8 agonist CL097 (23, 24) to activate freshly isolated neutrophils and screened the neutrophil expression of ligands to activating and inhibiting NKRs using flow cytometry. As demonstrated in Figure 1A, neutrophils stimulated with CL097 and GM-CSF for 20 min at 37°C displayed strong up-regulation of CD11b and CD66, both classical markers for neutrophil activation, as well as loss of CD62L. In parallel, we observed a significant decrease of classical HLA class I molecules after 20 min stimulation at 37°C (HLA-ABC; Figure 1A). These results were confirmed with two additional clones recognizing HLA-ABC (G46-2.6 and A6-136; data not shown). Notably, a significant downregulation of HLA class I was seen also without addition of stimuli (Figure 1A). Furthermore, we observed decreased expression of the DNAM-1 ligand PVR, and increased expression of MICA/B and ULBP-3, both ligands to NKG2D, on the neutrophil surface after 20 min of stimulation with CL097/GM-CSF (Figure 1B), while no robust changes were observed regarding expression levels of Nectin-2, ULBP-1, ULBP-2/5/6, B7-H6, or NKp46-ligand, all ligands to activating NKRs (Supplemental Figure 1). Surface expression of CD48, a ligand to 2B4, could not be detected on neither resting nor activated neutrophils (data not shown). Extended GM-CSF and TLR stimulation further decreased neutrophil surface expression of classical HLA class I molecules, while expression levels of ligands to activating NKRs displayed varying patterns (Figure 1C, Supplemental Figure 1).

**Figure 2:**
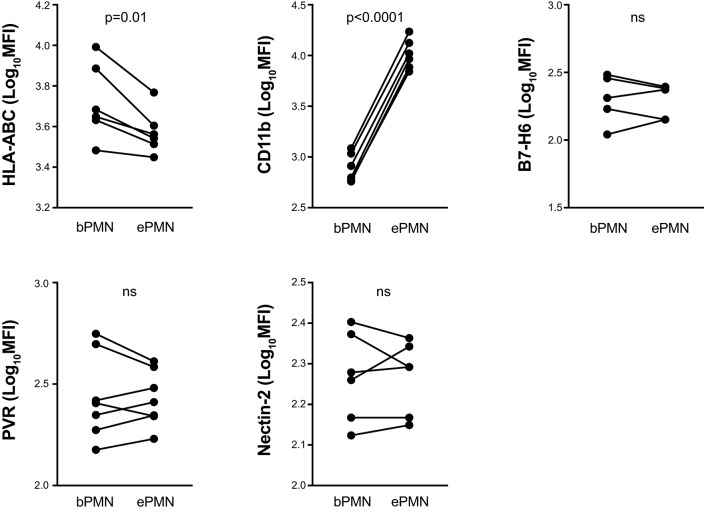
Surface expression of ligands to inhibitory and activating NKRs on transmigrated neutrophils. Surface staining of indicated ligands to NKRs on transmigrated neutrophils (ePMN), collected from exudates in the skin chamber model, and autologous blood neutrophils (bPMN). Paired *t*-test, error bars represent SEM.

To investigate whether our observations in neutrophils activated *in vitro* were consistent with expression patterns on activated, extravasated neutrophils *in vivo*, we made use of a human *in vivo* model where transmigrated neutrophils are harvested from a serum-filled skin chamber placed on exposed dermis (25). Transmigrated neutrophils were highly activated as reflected by an increased CD11b expression and loss of CD62L compared to resting autologous neutrophils harvested from blood (Figure 2 and data not shown). Similar to the *in vitro*-activated neutrophils, transmigrated neutrophils displayed significantly decreased surface expression of classical HLA class I molecules. Consistent with our data from *in vitro*-stimulated neutrophils, transmigrated neutrophils did not display altered surface expression of Nectin-2 and B7-H6. However, in contrast to *in vitro-*stimulated neutrophils, transmigrated neutrophils did not display decreased expression of PVR.

### Downmodulation of NK Cell-Regulatory HLA Molecules on Activated Neutrophils

**Figure 3:**
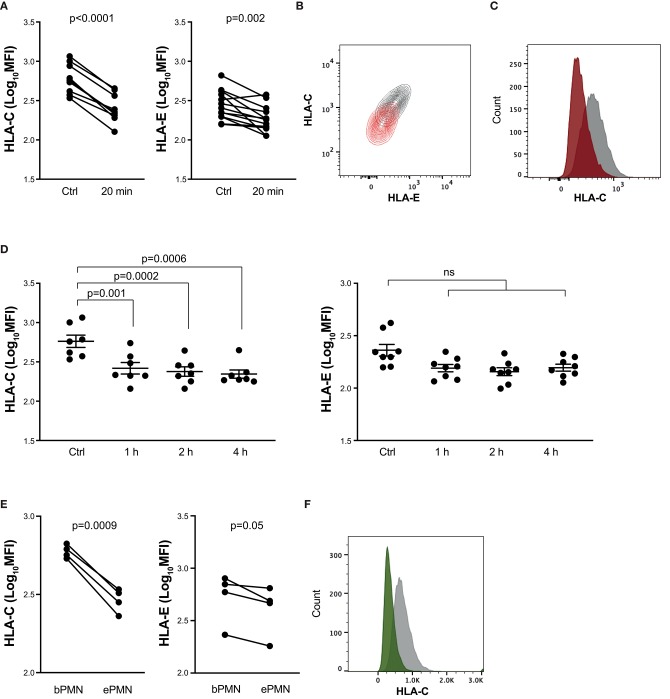
Downmodulation of HLA-C and HLA-E on activated neutrophils. **(A–D)** Neutrophil staining of indicated HLA class I cell surface structures (HLA-C or HLA-E) binding to inhibitory NKRs. Resting neutrophils were kept on ice (ctrl) or stimulated for 20 min with the TLR-agonist CL097 and GM-CSF at 37°C (**A**; paired *t*-test) or for 1, 2, or 4 h under the same conditions (**D**; one-way ANOVA followed by Dunnett's multiple comparisons test). **(B)** Representative staining of HLA-C and HLA-E of resting neutrophils (gray) or neutrophils that had been stimulated with CL097/GM-CSF for 20 min (orange). **(C)** Representative staining from one experiment showing expression of HLA-C on resting neutrophils (gray) or neutrophils that had been stimulated with CL097/GM-CSF for 1 h (red). **(E)** Neutrophil staining of indicated HLA class I molecules on transmigrated neutrophils from skin chamber exudates (ePMN) or resting autologous neutrophils (bPMN; paired *t*-test). **(F)** Representative staining from one experiment showing the expression of HLA-C on resting neutrophils (gray) or transmigrated neutrophils (green). Error bars represent SEM.

HLA class I expression on hematopoietic cells is dominated by HLA-A and -B, which are approximately 15 times more abundant than HLA-C (26), which in turn is more abundant than the non-classical HLA class I molecule, HLA-E. Given the important role for HLA-C and HLA-E in regulation of NK cell effector functions (27), we next investigated to what extent these HLA molecules were also downregulated in response to neutrophil stimulation. As shown in Figures 3A,B, we observed a strong downregulation of both HLA-C and HLA-E already after 20 min of stimulation. The decreased HLA-C expression was observed also after longer neutrophil stimulation (Figures 3C,D; Supplemental Figure 2). Consistently, we observed a similar picture on transmigrated neutrophils *in vivo* (Figures 3E,F).

**Figure 4:**
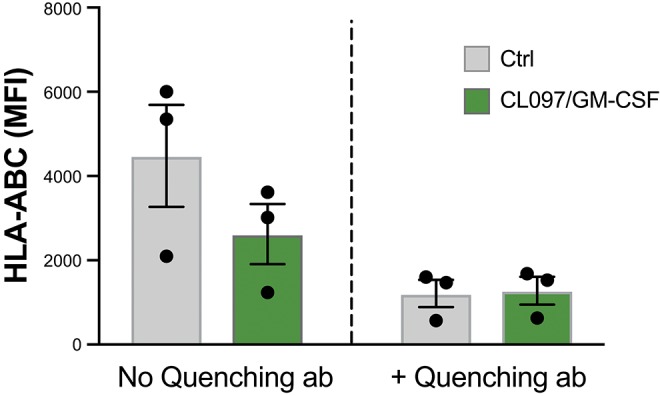
Decrease of surface HLA class I molecules is not explained by internalization. Graph shows resting neutrophils stained with FITC-conjugated HLA-ABC antibody prior to addition of buffer (ctrl) or CL097/GM-CSF for 20 min at 37°C, after which FITC-quenching antibody was added.

In an attempt to understand the mechanism of HLA downmodulation, we investigated whether HLA was internalized upon neutrophil activation. Neutrophils were stained with a FITC-conjugated anti-HLA-ABC antibody prior to stimulation. After 20 min CL097/GM-CSF stimulation, an anti-FITC mAb was added, quenching the extracellular FITC signal. However, as shown in Figure 4, the fluorescence detected from activated neutrophils was quenched to a similar extent as resting neutrophils, suggesting that internalization of HLA is not the main explanation to the HLA downregulation observed on activated neutrophils. Activated neutrophils release granules containing various proteolytic enzymes, such as serine proteases, e.g., elastase, proteinase 3, cathepsin G, and different matrix metalloproteases, and we next addressed to what extent inhibition of specific enzymes could restore HLA expression in activated neutrophils. However, Batimastat, an inhibitor of matrix metalloproteases, did not affect the neutrophil surface expression of HLA class I and neither did α1-antitrypsin nor specific inhibitors of cathepsin G and PR3 (data not shown).

### Reduced HLA Class I Expression on Activated Neutrophils Translates Into Enhanced Sensitivy to NK Cell Cytotoxicity

**Figure 5:**
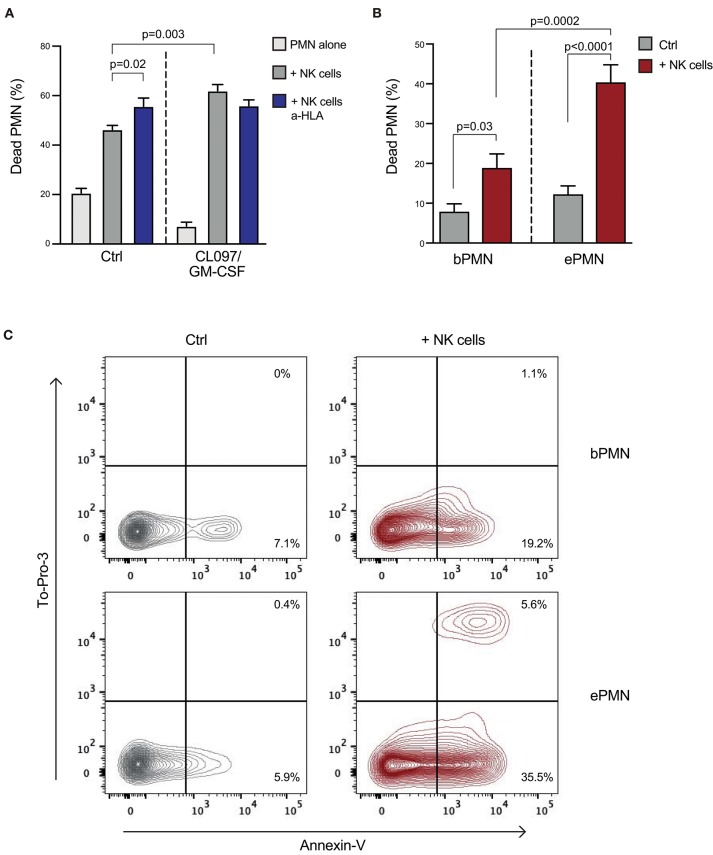
Activated neutrophils are more sensitive to NK cell cytotoxicity compared to resting neutrophils. **(A)** Percentage of dead neutrophils (PMN) after a 3 h co-culture of neutrophils [pre-stimulated *in vitro* with CL097 and GM-CSF, or kept on ice (ctrl)] and bulk-NK cells at an E:T ratio of 10:1, with addition of an HLA antibody as indicated (*n* = 7; one-way ANOVA followed by Dunnett's multiple comparisons test). **(B)** Graph shows percentage of dead neutrophils after a 4 h cytotoxicity assay performed using autologous NK effector cells toward transmigrated (ePMN) or blood neutrophils (bPMN; E:T ratio 10:1; *n* = 5; one-way ANOVA followed by Dunnett's multiple comparisons test). Error bars represent SEM. **(C)** Representative FACS plots showing percentage of dead neutrophils (transmigrated or resting) measured using AnnexinV and To-Pro-3, after a 4 h co-culture with autologous NK cells.

As activated neutrophils displayed lower expression of ligands to inhibitory NKRs, and no or modest modulation of their expression of ligands to activating NKRs was detected, we speculated that the outcome of interactions between NK cells and activated vs. resting neutrophils would differ. To investigate the neutrophil sensitivity to NK cell cytotoxicity, the viability of resting or pre-activated neutrophils was determined after co-culture with pre-activated NK cells. As demonstrated in Figures 5A,B, both transmigrated skin chamber neutrophils and *in vitro*-stimulated neutrophils were more sensitive to NK cell cytotoxicity compared to resting neutrophils, as reflected by increased binding of Annexin V to extracellular phosphatidyl serine and nuclear staining of cells with compromised plasma membrane integrity with To-Pro-3 (Figure 5C). Notably, addition of an HLA antibody, blocking the inhibitory interaction between HLA and KIRs or NKG2A/CD94, resulted in a significantly higher NK cell cytotoxicity against resting neutrophils (Figure 5A). By contrast, the cytotoxicity against activated neutrophils, which display low expression of HLA class I molecules, did not further increase upon HLA class I blockade. Blocking of NCR signaling *via* NKp46 resulted in decreased NK cell-mediated killing of *in vitro*-activated neutrophils (Supplemental Figure 3), consistent with our previous findings for resting neutrophils (6).

## Discussion

After executing their tasks in peripheral tissues, neutrophils, with their cargo of toxins and degrading enzymes, must undergo apoptosis to prevent prolonged inflammation and resulting tissue damage. During the last decade, a growing pile of evidence has identified an important role for NK cells as modulators of the immune response (21, 22, 24) and we have earlier demonstrated that NK cells can induce apoptosis in neutrophils in an NKp46-dependent manner (6). The outcome of NK cell—target cell interactions is dependent not only on cell signaling *via* activating receptors, but also on signaling *via* inhibitory receptors. In this study we investigated the neutrophil surface expression of ligands to inhibitory, as well as activating NKRs, on both *in vitro*-activated neutrophils and *in vivo*-transmigrated neutrophils isolated using a skin chamber model (25). With the exception of certain NKG2D ligands, the expression of ligands to activating NKRs remained largely unchanged upon neutrophil activation. By contrast, we observed a substantial downregulation of HLA class I molecules on activated neutrophils, which included also less abundant HLA structures of importance for NK cell regulation, HLA-C, and HLA-E.

HLA downregulation is a commonly used immunoevasive strategy among virus-infected cells or tumor cells to avoid T cell recognition and elimination. However, the decreased HLA expression will reduce inhibitory signaling in NK cells and thus allow missing-self recognition by NK cells (27–29). The dramatic downregulation of HLA class I molecules observed in this study necessarily involves downregulation of the abundant HLA subtypes, HLA-A and -B which according to previous reports comprise 95% of total HLA class I (26). Interestingly, our analysis demonstrated that the downregulation of HLA class I on activated neutrophils was not confined to the most abundant HLA class I molecules but also included pronounced downregulation of HLA-C and HLA-E. As HLA class I molecules act as ligands to inhibitory NK cell receptors, we hypothesized that the reduced surface expression of NK cell-specific HLA-C and HLA-E would result in higher sensitivity to NK cell cytotoxicity in inflammatory neutrophils. Indeed, the loss of surface HLA class I molecules rendered inflammatory neutrophils more susceptible to NK cell-mediated killing.

Our data suggest that HLA downregulation on inflammatory neutrophils may serve the purpose to increase their susceptibility to NK cell cytotoxicity and thus contribute to resolution of the inflammatory process by NK-induced neutrophil apoptosis. Chronic inflammatory diseases, such as rheumatoid arthritis, are characterized by an ongoing inflammation and presence of high numbers of inflammatory neutrophils (30). A focus of further studies should be to what extent aberrant HLA expression patterns or absence of certain NK cell phenotypes may be related to disease grade and characteristics. In this regard, it has been reported that the immature, less cytotoxic CD56^bright^ NK cells predominate in the inflamed synovium (31). This finding is in line with our data, as the less cytotoxic CD56^bright^ NK cell population may rather fuel the inflammatory process by producing inflammatory cytokines than kill inflammatory neutrophils.

We were not able to pinpoint the exact mechanism for neutrophil HLA downregulation in this study. It is possible that HLA molecules are internalized upon neutrophil activation. To test this hypothesis, we made use of an anti-FITC antibody, which quenches the FITC. As the neutrophils were stained with FITC-conjugated anti-HLA-ABC antibody before priming, internalization would mean that also the FITC-conjugate was internalized. The quenching antibody can only bind to extracellular FITC, and thus, any internal FITC signal would still fluoresce when hit by light in the right wave length. However, the FITC signal was not higher in activated neutrophils than in resting neutrophils, and we could thus not conclude that HLA was internalized. Alternatively, HLA molecules could be shed from the cell surface. Neutrophil granules contain enzymes, such as the serine proteases, neutrophil elastase, cathepsin G, and proteinase 3, which may be released upon neutrophil transmigration to an inflammatory site (32, 33). Shedding of surface B7-H6 from tumor cells has previously been described as a cause of matrix metalloprotease-mediated shedding (34), and presence of a soluble form of B7-H6 has been reported in supernatants from *in vitro*-stimulated neutrophils (35). This suggests that also neutrophil modulation of HLA molecules may involve proteolytic shedding. Supporting this hypothesis, the metalloprotease inhibitor Batimastat has been reported to inhibit release of soluble HLA class I in activated lymphocytes (19). In this study, however, we were unable to inhibit HLA class I downregulation on neutrophils with Batimastat, although the Batimastat readily inhibited shedding of CD62L from the activated neutrophils. Notably, a fraction of serine proteases from azurophil granules remain associated with the neutrophil cell membrane after granule-mobilization (33), and there are reports suggesting that membrane-bound forms of these enzymes are relatively insensitive to protease inhibitors (36). Thus, the inability of protease inhibitors to prevent HLA class I downregulation does not preclude a role for these proteases in this process.

A third alternative for the observed downregulation of HLA expression could be that neutrophil activation leads to a change in the HLA conformation, disabling antibody recognition of HLA. Several studies have reported open conformers of HLA, with the conformation change leading to a shift in receptor—ligand interactions (37–39). Surface staining of resting and activated neutrophils using an antibody recognizing an open conformation of HLA class I did not reveal any increase in HLA class I open conformers on activated neutrophils (data not shown). Nevertheless, in the study we observed HLA class I downregulation on activated neutrophils using five different antibody clones detecting HLA molecules (clones G46-2.6, W6/32 and A6-136 against HLA-ABC, clone DT9 against HLA-C, and clone 3D12 against HLA-E) strongly suggesting that HLA class I is decreased on the surface of inflammatory neutrophils.

In conclusion, we have demonstrated that both *in vitro*-activated and *in vivo*-transmigrated neutrophils display decreased surface expression of HLA class I molecules. In line with the decreased HLA class I expression, inflammatory neutrophils were more sensitive to NK cell-mediated cytotoxicity, as the loss of inhibitory signaling tips the balance toward NK cell activation and cytotoxicity. Collectively, our study demonstrates that neutrophils dynamically modulate their expression and release of NK cell receptor ligands, and that NK cells may contribute to a controlled resolution of the inflammatory process by killing activated neutrophils.

## Materials and Methods

### Antibodies and Reagents

Following mAbs were used for detection of surface markers: anti-CD11b APC (clone: ICRF44), CD48 BV421 (TÜ145), HLA-ABC FITC (G46-2.6), CD62L APC (DREG-56), CD66 PE (B1.1; all from BD Biosciences, CA, USA), HLA-C (DT9, Merck Millipore), HLA-ABC Alexa Fluor 647 (W6/32) and HLA-E PE-Cy7 (3D12; both from Biolegend), B7-H6 (875002), ULBP1 PerCP (170818), ULBP-3 PE (166510), ULBP-2/5/6 APC (165903; all from R&D Systems, MN, USA), CD56 PE-Cy7 (N901, Beckman Coulter, IN, USA), MICA/B viobright FITC (6D4, Miltenyi Biotech, Bergisch Gladbach, Germany), and Alexa Fluor 488-conjugated goat anti-mouse IgG (Invitrogen, CA, USA). Anti-B7-H6 (17B1.3) was kindly provided by Prof E Vivier (CMIL, Marseille, France), and anti-PVR (CD155; M5A10) and Nectin-2 (CD112; L14) were received from the lab of Prof. A Moretta (Genova, Italy). Goat serum and α1-antitrypsin were obtained from Sigma-Aldrich (MO, USA), CL097 from InvivoGen (San Diego, CA), GM-CSF from R&D Systems (MN, USA), anti-PR3 from Abcam (UK), and Batimastat and cathepsin G inhibitor from Calbiochem (CA, USA). In experiments where the extracellular FITC signal was quenched, a Fluorescin/Oregon green polyclonal antibody (ThermoFisher, Sweden) was used.

### Isolation of Human Leukocytes

Buffy coats or fresh blood obtained from healthy donors were mixed 1:1 with 2% dextran. After sedimentation of erythrocytes, granulocytes and PBMCs were separated by density gradient centrifugation (40, 41). Remaining erythrocytes were lysed in distilled water to obtain a pure granulocyte population. This method provides a granulocyte population of >95% purity. NK cells were isolated from PBMCs using an NK cell isolation kit (Miltenyi Biotec, Bergisch Gladbach, Germany), according to the manufacturer's protocol. To obtain activated, polyclonally expanded NK cells (bulk), freshly isolated NK cells were cultured on irradiated feeder cells in the presence of 100 U/mL recombinant human IL-2 (Proleukin; Chiron) and 1.5 ng/ml phytohemagglutinin (PHA) (GIBCO Ltd.).

### Isolation of Transmigrated Neutrophils

In order to isolate *in vivo*-transmigrated neutrophils, a skin chamber technique was used, described in detail elsewhere (25, 42). In short, blisters were introduced on the forearm on healthy volunteers using negative pressure. After 2 h, the blister roofs were removed and collection chambers were placed on top of the lesions. Autologous serum was added to the chamber wells. After 24 h, the chambers were removed and *in vivo*-transmigrated neutrophils were harvested from the skin chamber fluid, with a granulocyte purity >90%, and with mainly viable neutrophils (42). Autologous blood neutrophils were isolated from peripheral blood as described above and used as control cells. All neutrophils were diluted in Krebs Ringer Glucose (KRG) supplemented with Ca^2+^, and stored on melting ice until use. The study was approved by the regional ethical board in Gothenburg (543-07) and voluntary donors gave written informed consent in accordance with the declaration of Helsinki.

### Generation of Fusion Proteins for Detection of NKp46 Ligand

HEK293T cells, transfected with a construct encoding for a fusion protein consisting of the extracellular part of NKp46 fused to an Fc-portion of human IgG, were kindly provided by Prof. O Mandelboim (Jerusalem, Israel). Details of the transfection procedure has been described elsewhere (43, 44). Cells were cultured in DMEM (Sigma-Aldrich) supplemented with 10% heat inactivated fetal calf serum (FCS), 1% PEST, 1% L-glutamine and Puromycin dihydrochloride (5 μg/ml; Sigma-Aldrich). Cell cultures were kept at 37°C in a humidified, 5% CO_2_ atmosphere. When reaching 70% confluency, medium was exchanged to low protein medium supplemented with non-essential amino acids, 1% Penicillin-Streptomycin, 1% L-glutamine and 1% Sodium Pyruvate. After 3 days, the cell-free supernatant was collected and fusion proteins were purified with affinity chromatography using a HiTrap Protein G column (GE Healthcare). Proteins were eluted with 0.1 M Glycine/HCl (pH 2,7-3), neutralized in 1 M TRIS/HCl (pH 8), concentrated using an Amicon Ultra 15 centrifugation tube (Millipore) and stored in buffered NaCl in −20°C until use.

### Expression of Surface Ligands on Neutrophils

Freshly isolated neutrophils were stimulated with CL097/GM-CSF (1 μg/ml and 100 U/ml, respectively), at 37°C for indicated time. In some experiments, the matrix metalloproteinase (MMP) inhibitor Batimastat (10 μM), α1-antitrypsin (1 μg/ml), cathepsin G inhibitor (1 μM) or anti-PR3 antibody (100 ng/ml) were added to the cells. Supernatants were collected after priming for detection of soluble HLA. Cells were stained with antibodies for 30 min at 4°C. Degranulation/activation was monitored using antibodies directed against the surface markers CD11b, CD62L, and CD66. For detection of unconjugated antibodies (HLA-C, B7-H6, PVR and Nectin-2), cells were saturated in 20% goat serum for 10 min prior to incubation with Alexa Fluor-488 conjugated goat anti-mouse IgG. Samples were collected on a three-laser FACSAria (405, 488, and 633 nm) and data was analyzed using FlowJo software (TreeStar; version 9.7.2 or later).

For detection of NKp46 ligand, neutrophils were treated with the streptoccal enzyme EndoS, kindly provided by Dr. Mattias Collin (Lund University, Sweden), at 37°C for 20 min to digest endogenous IgG bound to the surface of the cells (45). Cells were subsequently incubated with fusion protein (10 μg/ml) for 30 min at 4°C. Cells were saturated in 20% goat serum prior to staining with FITC-conjugated Fcγ-specific goat anti-human IgG (Jackson Immunoresearch Laboratories, PA, USA).

### Detection of NK Cell-Induced Neutrophil Apoptosis

Freshly isolated neutrophils were stimulated with CL097/GM-CSF (1 μg/ml and 100 U/ml, respectively), or kept at 4°C without stimuli, for 1 h. After stimulation the neutrophils were co-cultured with polyclonal IL-2 activated NK cells at a 10:1 E:T ratio for 3 h at 37°C in the presence or absence of anti-HLA-I mAb (clone A6136, IgM), or anti-NKp46 (clone KL247, IgM). Transmigrated neutrophils collected from skin chamber exudates or autologous neutrophils isolated from blood were co-cultured with autologous NK cells, stimulated in IL-12 (1 ng/ml) or IL-15 (10 ng/ml) overnight. Apoptosis and lysis of neutrophils were detected with FITC-conjugated AnnexinV (BD Biosciences, CA, USA) and To-Pro-3 (Invitrogen) using flow cytometry (46).

### Statistics

For single pairwise comparisons, paired *t*-test was used. For multiple comparisons within a data set, one-way ANOVA followed by Dunnett's multiple comparisons test, was used.

## Data Availability Statement

The raw data supporting the conclusions of this manuscript will be made available by the authors, without undue reservation, to any qualified researcher.

## Ethics Statement

The studies involving human participants were reviewed and approved by The Regional Ethics Board in Gothenburg. The patients/participants provided their written informed consent to participate in this study.

## Author Contributions

EB, KC, SP, and FT designed the research. EB, KC, SP, and MP performed the research and analyzed the data. EM, SS, and FT supervised the study. EB, KC, and FT drafted the manuscript. All authors contributed to the final version of the manuscript.

### Conflict of Interest

The authors declare that the research was conducted in the absence of any commercial or financial relationships that could be construed as a potential conflict of interest.
