# Supplementary material for: Downregulation of HLA Class I Renders Inflammatory Neutrophils More Susceptible to NK Cell-Induced Apoptosis
Source: Front Immunol. 2019 Oct 15;10:2444. doi: 10.3389/fimmu.2019.02444 (PMC6803460; doi:10.3389/fimmu.2019.02444)
Supplement: Supplementary file 1 [file Data_Sheet_1.PDF]

Supplementary material

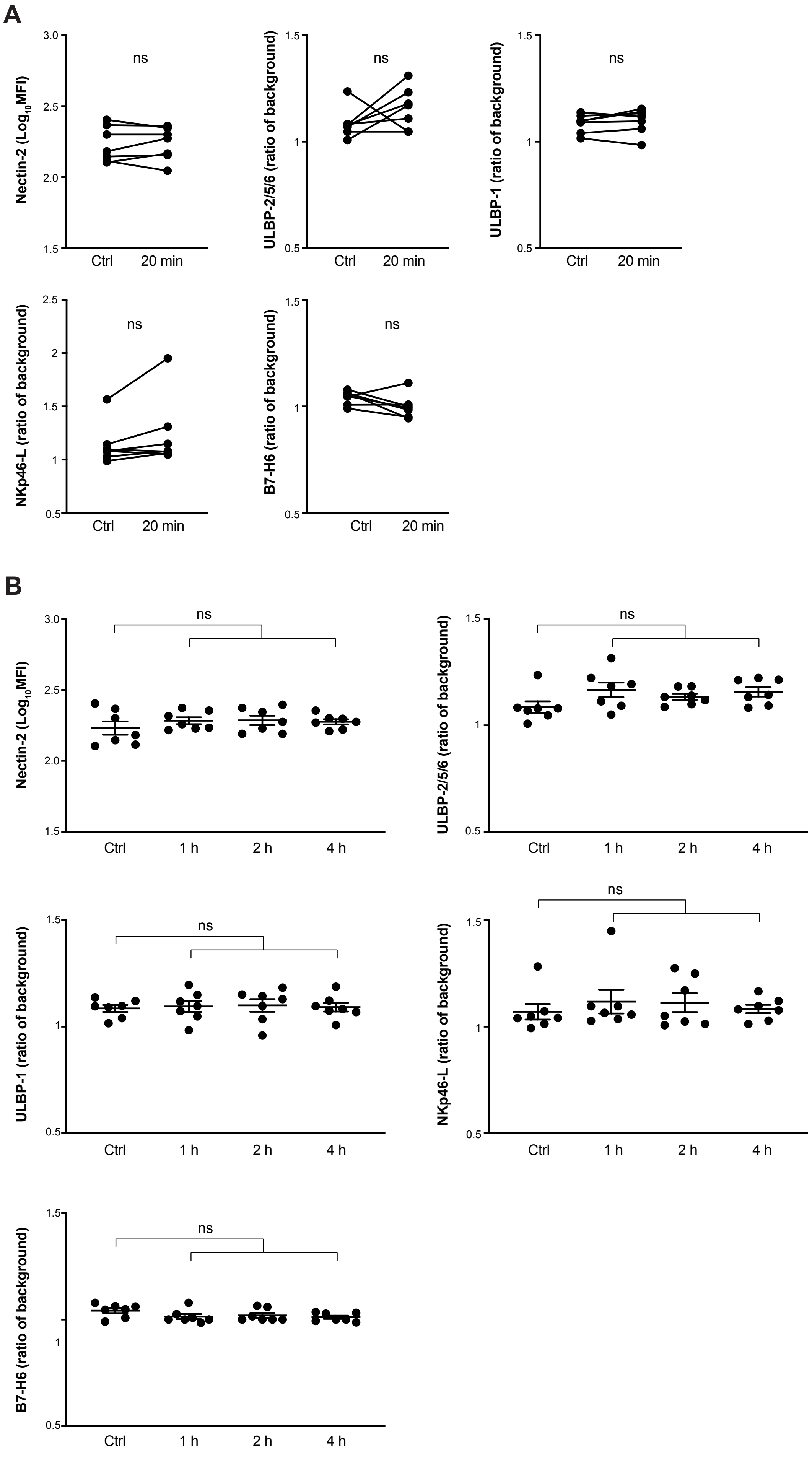

Supplemental Figure 1. **NKR ligand expression on *in vitro* activated neutrophils.** Surface expression of indicated ligands to NKRs on resting neutrophils (ctrl) or *in vitro* activated neutrophils stimulated at 37°C with CL097/GM-CSF for 20 min (**A**; paired t-test) and for 1, 2 or 4 hours (**B**; one-way ANOVA followed by Dunnett's multiple comparisons test). For NKp46-L staining, control cells were kept at 37°C. Error bars represent SEM.

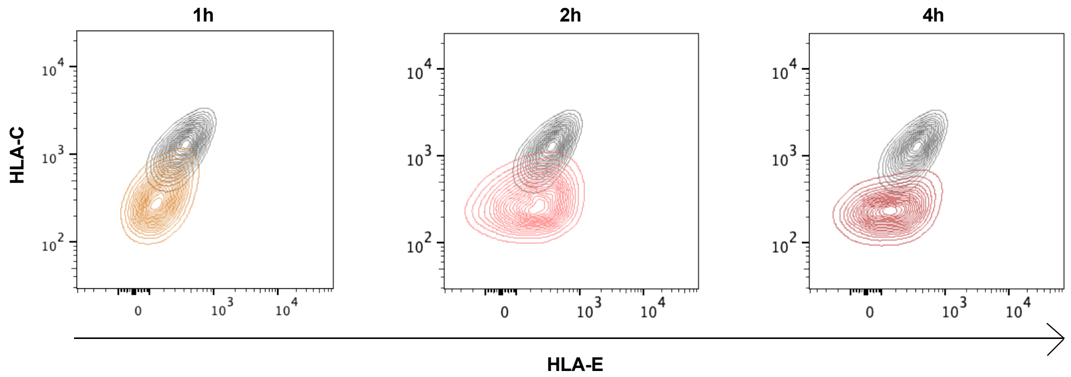

Supplemental Figure 2. Representative staining of HLA-C and HLA-E of resting neutrophils (grey) or neutrophils that had been stimulated with CL097/GM-CSF for 1 h (orange), 2 h (red) or 4 h (dark red) at 37°C.

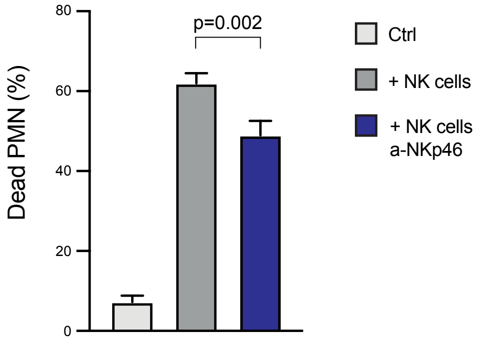

Supplemental Figure 3. Percentage of dead neutrophils (PMN) after a 3 h co-culture of neutrophils (pre-stimulated *in vitro* with CL097 and GM-CSF) and bulk-NK cells at an E:T ratio of 10:1, with addition of an NKp46 antibody as indicated (one-way ANOVA followed by Dunnett's multiple comparisons test). Error bars represent SEM.
